# Nanodelivery Systems Targeting Epidermal Growth Factor Receptors for Glioma Management

**DOI:** 10.3390/pharmaceutics12121198

**Published:** 2020-12-10

**Authors:** Sathishbabu Paranthaman, Meghana Goravinahalli Shivananjegowda, Manohar Mahadev, Afrasim Moin, Shivakumar Hagalavadi Nanjappa, Nandakumar Dalavaikodihalli Nanjaiyah, Saravana Babu Chidambaram, Devegowda Vishakante Gowda

**Affiliations:** 1 Department of Pharmaceutics, JSS College of Pharmacy, JSS Academy of Higher Education and Research, Mysuru 570015, India; psathishbabu@jssuni.edu.in (S.P.); gsmeghana@jssuni.edu.in (M.G.S.); mmanohar@jssuni.edu.in (M.M.); 2 Department of Pharmaceutics, Hail University, Hail PO BOX 2440, Saudi Arabia; a.moinuddin@uoh.edu.sa; 3 Department of Pharmaceutics, KLE College of Pharmacy, Bangalore 590010, India; shivakumarhn@gmail.com; 4 Department of Neurochemistry, National Institute of Mental Health and Neuroscience, Bangalore 560029, India; nandakumardn@nimhans.ac.in; 5 Department of Pharmacology, JSS College of Pharmacy, JSS Academy of Higher Education and Research, Mysuru 570015, India; saravanababu.c@jssuni.edu.in

**Keywords:** glioblastoma, receptor tyrosine kinases, epidermal growth factor receptor, small molecule inhibitors, nanoformulations

## Abstract

A paradigm shift in treating the most aggressive and malignant form of glioma is continuously evolving; however, these strategies do not provide a better life and survival index. Currently, neurosurgical debulking, radiotherapy, and chemotherapy are the treatment options available for glioma, but these are non-specific in action. Patients invariably develop resistance to these therapies, leading to recurrence and death. Receptor Tyrosine Kinases (RTKs) are among the most common cell surface proteins in glioma and play a significant role in malignant progression; thus, these are currently being explored as therapeutic targets. RTKs belong to the family of cell surface receptors that are activated by ligands which in turn activates two major downstream signaling pathways via Rapidly Accelerating Sarcoma/mitogen activated protein kinase/extracellular-signal-regulated kinase (Ras/MAPK/ERK) and phosphatidylinositol 3-kinase/a serine/threonine protein kinase/mammalian target of rapamycin (PI3K/AKT/mTOR). These pathways are critically involved in regulating cell proliferation, invasion, metabolism, autophagy, and apoptosis. Dysregulation in these pathways results in uncontrolled glioma cell proliferation, invasion, angiogenesis, and cancer progression. Thus, RTK pathways are considered a potential target in glioma management. This review summarizes the possible risk factors involved in the growth of glioblastoma (GBM). The role of RTKs inhibitors (TKIs) and the intracellular signaling pathways involved, small molecules under clinical trials, and the updates were discussed. We have also compiled information on the outcomes from the various endothelial growth factor receptor (EGFR)–TKIs-based nanoformulations from the preclinical and clinical points of view. Aided by an extensive literature search, we propose the challenges and potential opportunities for future research on EGFR–TKIs-based nanodelivery systems.

## 1. Introduction

Gliomas are the most common and lethal solid brain tumors and are known to affect about 0.02% of the worldwide population [1]. The occurrence of malignant gliomas and the frequency of cancer deaths have increased at an amplified rate across the world [2]. More than 330,000 new Central Nervous system (CNS) tumor cases and 227,000 brain cancer-related deaths were documented globally in the Global Burden of Disease (GBD) 2016 tumor database [3]. Despite the increase in cancer awareness programs, advancement in diagnostic tools, and treatment strategies in the United States, the prevalence of gliomas has been unstoppable [4,5,6].

Based on the molecular characteristics and origin of apparent cell types, the CNS tumors were classified using I to IV grade criteria by the World Health Organization (WHO) in 2007 and 2016 [7]. Accordingly, Grade, I, II, III, and IV are pilocytic astrocytomas, gliomas including diffuse astrocytomas, anaplastic astrocytomas, and glioblastoma multiforme (GBM), respectively [8,9]. The current standard treatment approaches across the world are dependent on surgery, radiotherapy, and chemotherapeutic drugs, i.e., temozolomide (TMZ), which resulted in the average survival rate of about 14 months [10,11,12]. Therefore, there is a definite need for understanding the molecular pathways and mechanisms involved in GBM pathology and thereby determine better management [13].

Generally, Receptor Tyrosine Kinase (RTKs) are commonly identified cell surface receptors that are considered to be pivotal regulators of critical cellular processes (epidermal growth factor receptor (EGFR) and vascular endothelial growth factor receptor (VEGFR)). EGFR is a transmembrane receptor tyrosine kinase that controls cancer cell proliferation, migration, differentiation, and homeostasis [14]. Nearly 50–60% of GBMs have EGFR genetic variants, with mutations, readjustments, selective linking, and amplification [15].

Over the past decades, many investigators have hundreds of designs and synthesized small molecular drugs as RTK inhibitors with extensive research. The Food and Drug Administration (FDA) has approved a few medications as first-line therapy for various forms of cancer (Table 1) [16]. However, the significant development of anti-cancer components has developed new problems [17]. For example, clinical studies that were conducted for the first and second generation of anti-EGFR drugs on the inhibition of cell growth, angiogenesis, and proliferation were found to be of no therapeutic benefit in GBM treatment. Many researchers also reported significant limitations such as low solubility, poor oral bioavailability, and severe adverse effects in the existing EGFR–TKIs drugs. In addition, the gradual rise of drug resistance during therapy instantly needs to be addressed [18]. The third generation of EGFR–TKIs drug (AZD9291) was developed recently and confirmed to have an effective preclinical investigation in GBM the in vitro and in vivo models’ above-listed drawbacks [15]. The advantages of nanotechnology offer a potential drug delivery approach with apparent benefits of nanoformulations such as lesser particle size, bulky surface area, excellent surface reactivity, active sites, and appropriate adsorption ability. Nano particles (NPs) applied as drug transporters have the potential to increase drug absorption and bioavailability, enrich effective targeting delivery, prolong the circulation time, and limit the dangerous side effects on healthy tissues [19].

In the present review, the authors have summarized epidemiology and risk factors associated with GBM, RTKs, and their inhibitors of intracellular signaling pathways in glioma, the clinical profile of small molecule inhibitors (EGFR–TKIs) drugs, and the associated multiple failures/resistance. In addition, the current progressive research of various nanopreparations for EGFR–TKIs and the combination of chemotherapeutic drugs to target GBM have been discussed. Aided by an extensive literature search and review, the authors have also proposed the possibilities and challenges for upcoming research on EGFR-TKIs and other chemotherapeutic agents.

## 2. Molecular Pathology of Glioma

Different genetic investigations determine various noteworthy biomarkers. Many of these were utilized in neuro-oncology to identify glioma patients, specifically the combined losses of the chromosome arms 1p and 19q in oligodendroglial cancer. Then, the methylation status of O-6 methylguanine–DNA methyltransferase gene promoter and modifications in the EGFR pathway in GBM, isocitrate dehydrogenase 1 (IDH1) and IDH2 gene mutations in diffuse gliomas, as well as B-Raf status in pilocytic astrocytomas. These groups are associated with different prognosis, germline variants, and the median age at diagnosis, highlighting different pathogenic mechanisms [20]. Although most GBM patients receive standard treatments, significant variations in clinical outcomes are often seen due to the heterogeneity of the tumors [21,22]. Hence, it is essential to determine more significant and practical biomarkers for analyzing the prognosis in GBM patients. Inflammation and immunity are critically involved in glioma initiation and progression [23,24], and various study reports suggested that inflammatory response cells such as neutrophils [25], lymphocytes [26], and platelets [27] are associated with the prognosis of cancer patients. In recent years, the prognostic value of preoperative hematological markers, such as albumin-to-globulin ratio (AGR), monocyte-to-lymphocyte ratio (MLR), median platelet volume (MPV), neutrophil-to-lymphocyte ratio (NLR), platelet distribution width (PDW), and platelet-to-lymphocyte ratio (PLR), have been investigated in several cancers, including gliomas [28,29,30,31,32]. However, there were no scientific investigations to the prognostic value of hematological biomarkers in a cohort of gliomas, mainly in relation to the various molecular classes. Therefore, a study examined the predictive value of preoperative hematological biomarkers (AGR, MLR, MPV, NLR, PDW, and PLR) alone and in combination with the five glioma molecular groups on the clinical trial results of a comparatively great cohort (n = 592) of Grade II–IV GBM patients. Based on these results, we suggest an analytical model for Grade II–IV GBM based on molecular pathology and NLR, and identify for lower-grade gliomas (LGG) four risk groups with distinct overall survival [33].

## 3. Epidemiology and Risk Factors of Glioma

In the past few decades, the investigation of adult glioma was prioritized because of a lesser global incidence of GBM, i.e., 10 per 100,000 people. Due to the lack of new and efficient diagnostic strategies, the survival rate (SR) of 15 months after diagnosis creates a critical public health problem [2,34,35]. GBM accounts for 50% of all gliomas in various age groups [36]. Although the peak incidence is between 55 and 60 years of age, GBM could occur at any age, with a mortality rate of 2.5% of the worldwide cancer death toll. GBM accounts for the third foremost cause of deaths due to cancer in patients from 15 to 34 years [37,38]. The GBM incidence ratio was more in men when compared to females [39]. The Western world reported a higher incidence of gliomas than less developed countries, which could be recognized as due to under-recording glioma cases, narrow contact to health care, and alterations in diagnostic practices [40,41,42]. A few studies showed that blacks were less prone to GBM. Further, the incidence of GBM was reported to be higher in Asians, Latinos, and Whites [43].

The current global standard for the catalog and identification of gliomas is as per WHO classification. WHO categorizes gliomas as Grade I to IV based on malignancy level, which is committed by the histopathological measures. Class I to III gliomas relay to abrasions with less proliferative potential and can be managed surgically with chemo and/or radiotherapy. In contrast, Grade IV gliomas are highly malignant and invasive. GBM is the utmost aggressive, offensive, and identical type of cancer and was labeled as Grade IV [44].

A positive family history, absence of atrophic conditions, longer length of leukocyte telomere, and risk alleles at more than twenty genetic loci are a few of the endogenous factors that enhance glioma risk. A high dose of ionizing radiation is also one of the environmental factors attributed to a higher risk of glioma [45]. Identifying modifiable factors that would enable primary prevention approaches remain the quintessential goal of glioma epidemiologic research.

Several studies on the allergic and nutritional epidemiology of glioma showed an inverse association between allergy and gliomas but did not provide any causal relationship between them [46]. The nutritional epidemiology studies suggested that various food groups and nutrients were associated with glioma risk; the results were inconclusive and not replicable in subsequent research [24,47]. The results of epidemiological analysis also suggested the presence of an inverse association between cancer and certain neurological conditions, mainly age-related neurodegenerative diseases [48]. In recent research, a potent negative association was observed between the expression levels of microRNAs in GBM than Alzheimer’s disease (AD), suggesting that although the molecular pathways behind the development of these two pathologies are the same, they appear to be inversely controlled by microRNAs [49]. Another epidemiological study indicated that the patients suffering from AD have a lower risk of developing lung cancer (LC) and suggest a higher risk of developing GBM [50].

## 4. Receptors Tyrosine Kinase (RTK) and Their Inhibitors of Intracellular Signaling Pathways in Glioma

**Figure 1:**
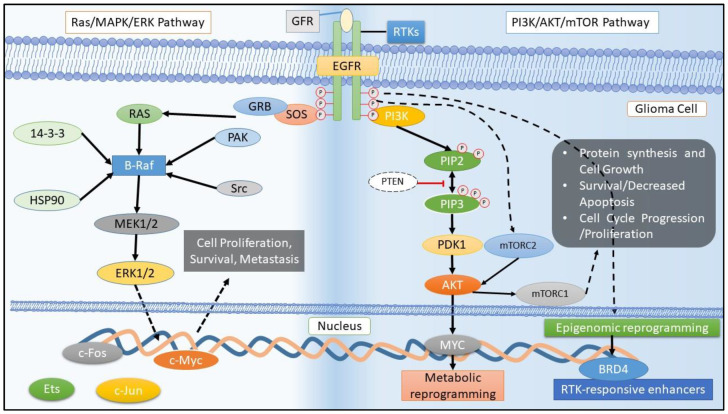
Schematic representation of Receptor Tyrosine Kinases (RTK) activation and the downstream signaling. RTKs, particularly epidermal growth factor receptors (EGFR), are amplified in glioblastoma, which significantly alters the nutrient uptake and utilization. The Rapidly Accelerating Sarcoma/mitogen activated protein kinase/extracellular-signal-regulated kinase (Ras/MAPK/ERK) and phosphatidylinositol 3-kinase/a serine/threonine protein kinase/mammalian target of rapamycin (PI3K/AKT/mTOR) pathways get activated through the stimulation by growth factor receptor (GFR). Physiologically, these two pathways orchestrate to execute cell proliferation, survival, motility, adhesion, and angiogenesis. Any deregulation in these pathways leads to an activation of oncogenic signaling cascades causing glioma.

RTKs belong to the family of cell surface receptors and are receptors for hormones, growth factors, neurotrophic factors, cytokines, and extracellular signaling molecules. The tyrosine kinase (TK) comprises the intracellular TK domain, extracellular ligand-binding domain, and a hydrophobic transmembrane domain. The domains as mentioned above get activated upon binding of the ligand, leading to the TK domain’s autophosphorylation and dimerization. This receptor, when activated by ligands in turn, activates two downstream signaling pathways, Rapidly Accelerating Sarcoma/mitogen activated protein kinase/extracellular-signal-regulated kinase (Ras/MAPK/ERK) and phosphatidylinositol 3-kinase/a serine/threonine protein kinase/mammalian target of rapamycin (PI3K/AKT/mTOR) [51] (Figure 1), which play a prominent role in cell differentiation, survival, proliferation, and angiogenesis. Thus, RTKs and their ligands were proven to be promising targets in the treatment of GBM. Among the several receptors belonging to the RTK group in human glioma, the signaling pathways such as EGFR and VEGF receptor mutations have played a significant role in GBM described below in detail [52].

### 4.1. EGFR Family and Its Mutations

RTKs that generally control the proliferation, migration, and differentiation of neural progenitors via signaling EGFR and its downstream MAPK and PI3K/AKT/mTOR pathways (Figure 1) [17]. EGFR, a member of the ErbB family, is commonly expressed in neural progenitors during brain growth and initiated stem cell astrocytes and transit-amplifying cells in the adult rodent subventricular zone (SVZ) [53,54]. Among 45–57% of GBM patients, the mutation and amplification in EGFR ErbB1 (EGFR, HER1) were detected, which indicated its major role in the pathogenesis of GBM [55,56]. In addition, about 8–41% of GBM patients showed mutations in erythroblastic oncogenic B/human epidermal growth factor receptor (ErbB2/HER-2) [55,57]. Its expression reduces significantly in the adult human cortex (Cx) and white matter (WM) under non-reactive conditions but is retained within the adult human SVZ astrocyte ribbon. The mechanisms maintaining more EGFR expression in human neural growth and its silencing upon difference are not well understood and not have been investigated before at the epigenetic level [58]. Excitingly, the most diffuse gliomas of LGG and high-grade glioma (HGG) have shown the pathological expression of EGFR. Generally, EGFR overexpression in gliomas has been mainly recognized to gene amplification, the activating mutation EGFRvIII, and gene fusion events, which overall comprise approximately half of GBM and are rarely observed in LGG [59,60]. EGFRvIII, a truncated species, is often expressed in GBM and independently activated by a ligand, resulting in cell survival and proliferation. Despite the growth-enhancing properties of the EGFRvIII, its expression has been linked to the increasing overall survival of patients. Furthermore, EGFRvIII, being a neoantigen, equally elicits an immune response [60,61]. Recent investigations have started to explore EGFR overexpression mechanisms in gliomas outside of genetic alterations, including the role of epigenetics. Still, there is no study that has analyzed the EGFR promoter in human glioma samples.

### 4.2. VEGF Family and Its Mutations

VEGF, a potent angiogenic protein, is known to enhance vascular permeability. Although VEGF has a role in normal tissues, malignant transformation has been shown to induce VEGF expression—especially under hypoxic conditions inducing the transcription factors (HIF1α and HIF1β) to translocate to the nucleus, thereby activating the VEGF gene [61] (Figure 1). Upon activation of the VEGF gene, angiogenesis is enhanced to neutralize the hypoxia. GBM tumors enhanced the expression of VEGF and hypoxia, which in turn caused irregular vasculature [62]. The enhanced expression of VEGF in GBM tissues was due to the up-regulation of the VEGF receptor, VEGFR2, which acted by RAS (Rapidly Accelerating Sarcoma) or PI3K (phosphatidylinositol 3-kinase) or the PLCγ–PKC–MAPK pathway in contrast to RTKs [63]. VEGFR3 operates similarly to TK activities. The PKC and RAS pathway is known to be stimulated by lymphangiogenesis in VEGFR-3. VEGF was also shown to play a vital role in vascularization and endothelial cells’ neoplastic growth [64].

## 5. Molecular Drug Therapy Targets and Its Clinical Profile of EGFR Family in Glioma

### 5.1. Small-Molecule Kinase Inhibitors

**Figure 2:**
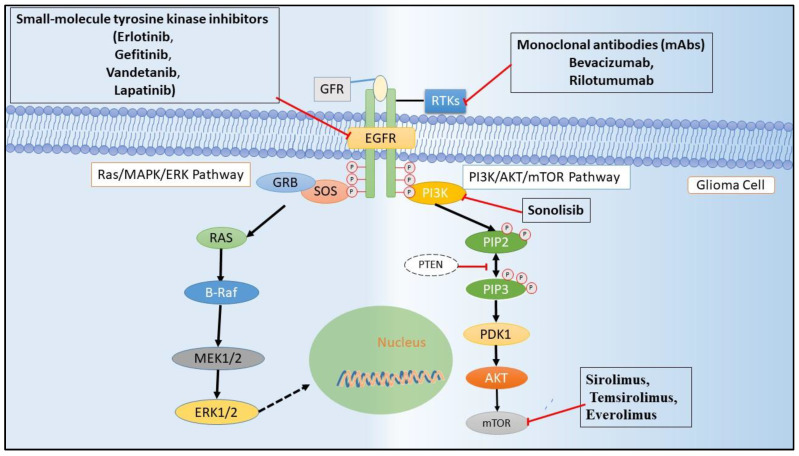
RTKs (EGFR) signal transduction and are a target site for small molecule and monoclonal antibody in glioma treatment. Small-molecule tyrosine kinase inhibitors block the downstream signaling by competing for ATP at the catalytic site of the kinase domain whilst the monoclonal antibodies (mAbs), which have an outstanding degree of specificity, block downstream signaling by binding to the leucine-rich and cysteine-rich ectodomains. Compounds that inhibit mammalian target of rapamycin (mTOR), a downstream signal in the EGFR pathway, facilitate the autophagic clearance of cancerous cells.

Various active components prevent the EGFR activity, and its ligands have been under progress since the starting of this era. Small molecular EGFR protein tyrosine kinase inhibitors (EGFR–TKIs) have become the most innovative active component in anti-cancer management [52]. EGFR–TKIs are a 4-anilinoquinazoline structure that could covalently link with the ATP binding site of the RTK to procedure the dynamic conformation. The initiation loop was phosphorylated and consequently inhibited the phosphorylation of TK (Figure 2) [65].

Erlotinib, an EGFR-TKI drug, prevents the phosphorylation of the TK intracellular domain of EGFR [66]. Several phase II studies for GBM were not efficient in recurrent GBM [67] patients. In contrast, Erlotinib’s combination therapy with temozolamide was well tolerated and enhanced the survival rate in the newly diagnosed GBM patients [68,69]. Gefitinib (ZD1839/Iressa^®^), another EGFR–TKI, radio sensitized U251 GBM cells in vitro [70]. Still, there was no improvement in the survival rate shown in the phase II clinical trial with newly diagnosed GBM patients [71]. AEE788 and Vandetanib inhibited both EGFR and VEGFR TK (Table 2), but when tested on GBM, patients showed lesser efficacy or enhanced toxicity. AEE788, in a phase I clinical trial, exhibited less efficacy and higher toxicity in treating recurrent GBM patients [72]. Although AEE788 showed very little efficacy in an in vitro GBM cell line, it decreased cell proliferation in vitro when administered in combination with histone deacetylase inhibitors (HDACIs) [73]. In a phase II trial, when incorporated into the standard regimen (surgery + chemotherapy + radiotherapy), AEE788 showed no/little effects, due to which the study was terminated [74].

Lapatinib, an inhibitor of both EGFR and HER2 TKs, showed little effect in a phase I/II clinical trial [75], but in combination therapy with CUDC-101, an HDAC inhibitor, it enhanced the radiosensitivity of the GBM cell line in vitro [76]. Few VEGFR–TKIs such as vatalanib (PTK787), sorafenib, and tivozanib showed lesser efficacy when individually administered (Table 2). Vatalanib and tivozanib did not affect the tumor volume; however, they were well tolerated in a phase II trial [77,78]. Sorafenib’s combination therapy with the standard regimen had little effect on recurrent GBM patients in a phase II trial [79]. A phase III trial of Cediranib (AZD2171), another VEGFR–TKI, failed to improve the progression-free survival, both in monotherapy and lomustine recurrent GBM patients [80].

### 5.2. Targeting Extracellular Domain of RTKs through Antibody Therapies

Among the various therapies targeted toward the kinase domains of RTK, the extracellular domain also served as a probable target for antibody therapy. The antibodies antagonized the ligand-binding site of RTKs, preventing the ligand binding and thereby activating the kinase domains [81]. An EGFR targeting the antibody cetuximab showed antagonistic activity by inhibiting the activation of RTKs, which in turn inhibited the tumor malignancy [82]. The antibody was used as rescue therapy in patients who have not responded to standard treatment. In addition, cetuximab monotherapy was well tolerated, and minimal recurrence of GBM was reported by a phase II clinical trial [83]. Another monoclonal antibody (mAb), ornartuzumab, targeting the hepatocyte growth factor receptor/tyrosine-protein kinase Met (HGFR/c-MET) receptor’s extracellular domain was reported to prevent the cancer growth in orthotopic U87 GBM xenograft. MK-0646 (H7C10/F50035/dalotuzumab), a humanized monoclonal insulin-like growth factor receptor type 1 (IGF-1R) antibody, was shown to be an antagonist that decreased cell proliferation and induced apoptosis [84].

The antibody therapies are still in the preliminary stages of investigation and are promising therapeutic targets for GBM compared to the small molecule kinase inhibitors [85,86]. In addition, the primary constraint faced in the antibody therapy was the Blood Brain Barrier (BBB) penetrability and the large size of molecules, which could be overcome by engineered antibodies capable of penetrating the BBB [87]. Antibodies binding with the transferrin receptors were used to cross the BBB in both murine and primate models. On the other hand, using Ommaya reservoirs or during surgery, the antibodies could directly be delivered to the brain, bypassing the BBB [88].

### 5.3. Therapies Directed at RTK Ligands

Antibodies not only bind to the extracellular domains but also are capable of trapping the ligands that activate the RTK signaling pathways [89]. Targeting the ligands might serve as an attractive means for GBM therapy. However, the usage of antibody was reduced due to various factors such as mutations in EGFRvIII and the inability to cross the BBB, which limited the tumor penetration and efficacy of the therapy. Bevacizumab, a humanized murine mAb, was reported to bind to VEGF and prevent it from binding to the receptor [90]. Bevacizumab was granted accelerated approval by the FDA in 2009; however, the drug demonstrated reduced efficacy against the newly diagnosed GBM and had no benefit on the patient’s overall survival [91]. Aflibercept, another trap for VEGF, prevented its binding to the receptor, and recurrent GBM patients were proven to have only 7.7% of participants resulting in progression-free survival rates after six months in a phase II trial. Rilotumumab (AMG102), an anti-HGF mAb, was shown to bind to HGF, thus preventing the binding to the HGFR/c-MET and thereby activating downstream targets. In combination with temozolomide in vitro, rilotumumab was shown to inhibit the growth of U87MG glioblastoma cells. The combination showed only minimal effects on GBM in the phase II clinical trial [18].

### 5.4. Targeting Downstream Pathway of EGFR

A comprehensively studied downstream pathway of EGFR is the PI3K/AKT/mTOR, which is critically involved in regulating cell apoptosis, autophagy, proliferation, and metabolism. Dysregulation of the pathway, as mentioned earlier, played a prominent role in various cancers [92,93]. Therapeutic strategies targeting PI3K/AKT in GBM have given promising results in the in vitro and in vivo xenograft models; however, clinical safety and efficacy need to be proven. Sonolisib (PX-866), an irreversible PI3K inhibitor, inhibited the angiogenesis and invasion of GBM cells in vitro but did not induce the apoptosis of GBM cells; nevertheless, the drug caused cell cycle arrest. Sonolisib was well tolerated but showed disease progression in almost 73% of recurrent GBM patients in the phase II clinical trial [94]. Various other inhibitors such as XL765 (SAR245409) and GDC-0084, both PI3K and mTOR inhibitors, showed efficacy against GBM in the in vitro and in vivo models. However, the results as mentioned above lack the support from relevant clinical data [95].

Sirolimus (rapamycin), temsirolimus (CCI-779), and everolimus (RAD001), the mTOR inhibitors were evaluated in the various clinical phases and showed little efficacy in treating GBM patients. Everolimus showed very little effectiveness and a low survival rate in monotherapy and combination with temozolomide radiotherapy in a phase II clinical trial [96]. Sirolimus monotherapy and in combination with erlotinib [97] and temsirolimus [98] failed to show any effect in the treatment of GBM patients in a phase II clinical trial.

Other inhibitors such as vistusertib (AZD2014), palomid 529, and mTOR kinase inhibitor (CC-223) were dual inhibitors of mTORC1 and mTORC2. Vistusertib showed radiosensitization in GBM cell lines both in the in vitro and in vivo models, due to which the participants were recruited to phase I and II clinical trials [99] (clinical trial ID: NCT02619864). In a GBM xenograft model (U87MG cells), CC-223 exhibited an anti-tumor effect, while Palomid 529 exhibited anti-tumor activity in the orthotopic murine tumor model [100].

## 6. Mechanism of Drug Resistance to EGFR–TKIs in Glioma

Although the mechanism of drug resistance to EGFR–TKI in GBM remain unclear, few reports discussed the possible mechanisms in this regard. The absence of mutation in exons 19 and 21 of the TK domain was reported, especially in first-line EGFR-TKIs such as erlotinib and gefitinib. Their pharmacological actions were dependent on the modifications, as mentioned above [101]. Another possible mechanism mentioned was an alternative activating signal that compensated for the inactivation of EGFR signaling by EGFR–TKIs. In addition, the absence of EGFRvIII and loss of phosphatase and tensin homolog (PTEN) were the other determinants of resistance in certain studies [102].

The inhibition of mTOR, a downstream molecule of the PI3K/PTEN/AKT pathway, promoted the response of glioma cells to EGFR-TKIs in vitro [103,104]. Conversely, there was no responsiveness to erlotinib and no expression of EGFRvIII and PTEN in the phase II clinical trial with relapsed GBM patients [105]. In addition, a combination of the mTOR and EGFR–TKIs inhibitors (sirolimus) did not improve the patients’ responsiveness in recurrent GBM patients [106]. On the other hand, erlotinib inhibited EGFR in EGFRvIII expressing U87 GBM cells and enhanced the expression of PDGFRα, thereby compensating the signaling pathway inhibited by erlotinib [97].

Despite the numerous studies on GBM treatment targeting EGFR, no therapeutic efficacy has been reported [107,108]. The therapeutic efficacy was minimal or nil in the case of first and second-generation EGFR inhibitors for the treatment of recurrent GBM [109,110]. The primary reasons for the above drugs’ failure were their inability to cross the BBB and the requirement of a relatively high amount of drug concentrations in the brain [92], which in turn limited their usage. By overcoming the above-said limitations, effective therapy for GBM could be discovered [92].

## 7. Current Pharmaceutical Drug Targets in Glioma

Despite the great activity of EGFR–TKIs, mAb, and chemotherapeutic agents, the therapeutic outcomes limited by BBB penetration in both preclinical and clinical studies have urged the thought of using TKIs-loaded nanoformulations in the management of GBM [111]. For example, lipophobic and less molecular weight drugs could not achieve specific delivery in tumor tissues and were characterized by a short circulation half-life [112]. Furthermore, compared to the other cancer types harboring EGFR amplification, clonal resistance was not observed in GBM after the EGFR inhibitor treatment. However, multiple failures and/or resistance such as the absence of exons 19 and 21 of the TK domain, an alternative activation of signals, rapid adaptive responses due to EGFR inhibitors, and the lesser ability of EGFR–TKI drugs to cross the BBB were reported in various studies [113].

**Figure 3:**
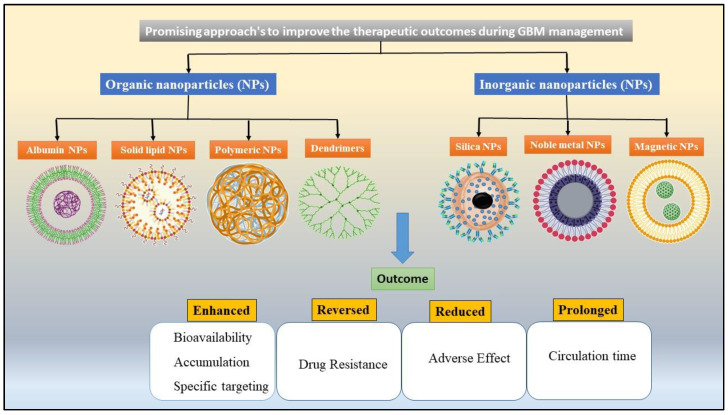
Different nanodelivery systems that facilitate BBB penetration and target-specific action in glioma.

Consequently, the novel drug delivery systems (NDDS) were employed for the specific delivery of FDA-approved drugs to increase its therapeutic outcomes and reduce the adverse effects during GBM treatment. Among the above-mentioned NDDS, the NPs (Figure 3) with various structures and properties to serve as the desirable carriers for anti-cancer drugs were invented [114,115]. In addition, the systems appear to be promising approaches to solve the existing problems in the management of GBM [114]. NPs-based systems have many unique benefits. Firstly, NPs can be loaded with the hydrophilic and hydrophobic drugs simultaneously, which results in an enhanced solubility and anti-cancer effect when employed with the suitable combinations of medicines in carriers. Secondly, the uniform particle size distribution and surface modifications enabled passive or active cancer targeting and resulted in improved drug availability in the tumor region. Lastly, the NPs as drug carriers also aided the sustained and controlled drug release at a specific region. The augmented drug release profiles with extended circulation time permitted improved pharmacokinetics and decreased the dose-dependent toxicity of therapeutic agents [116,117]. Hence, the subsequent portion reviews the benefits of various classes of NPs used in EGFR-targeted drug delivery to manage glioma.

### 7.1. Organic Nanoparticles

#### 7.1.1. Albumin Nanoparticles

**Figure 4:**
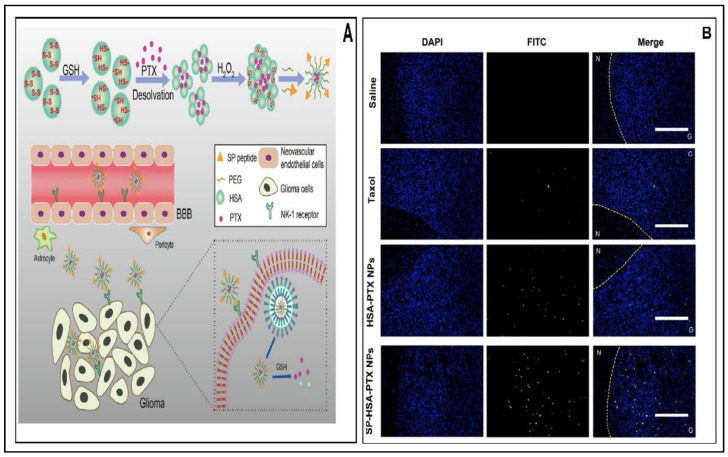
(**A**) Schematic representation of preparation and mechanism of action of albumin nanoparticles (NPs); (**B**) Fluorescent microscopic terminal deoxynucleotidyl transferase dUTP nick end labeling (TUNEL) assay images of in vivo anti-cancer efficacy of SP–HSA–PTX NPs (Green: TUNEL-stained apoptosis cells. Blue: 4′,6-diamidino-2-phenylindole (DAPI)-labeled nucleus, Yellow dashed lines: boundary between (N) normal brain and (G) glioma section). Reprinted with permission from [119], Elsevier, 2018.

Due to the greater biodegradability and low immunogenicity of serum albumin, it has been identified as a suitable nanocarrier for the cancer management in recent years. In addition, the ability of binding or absorbent proteins around the NPs was showcased as the foremost prominent factor for prolonged circulation time and phagocytosis [118]. As an endogenous substance, albumin might inhibit therapeutics drugs from unnecessary stability interaction and targeting efficiency. The human serum albumin-based paclitaxel (PTX) nanoparticles exhibited superior anti-tumor activity by the prolongation of survival and pro-apoptotic effect, as depicted by terminal deoxynucleotidyl transferase dUTP nick end labeling (TUNEL) analysis, thereby serving as a novel strategy for treating GBM (Figure 4A,B) [119]. Furthermore, radioiodine cross-linking anti-EGFR (cetuximab) and bovine serum albumin (BSA) polycaprolactone (PCL) nanoparticles were also useful to induce tumor regression, which in turn enhanced the cytotoxicity on tumor cells and limited the adverse effects of chemical agents [120]. Thus, it can be stated that albumin NPs exhibited an improvement in therapeutic outcomes.

Tsutsui et al. demonstrated bio-nano capsules (BNCs) as an efficient way to deliver drugs to brain tumors in Gli36 cell lines. BNCs are composed of a hepatitis B surface antigen, small interfering ribonucleic acid (siRNA), genes, chemical components, and proteins that selectively target brain tumors. BNCs, when conjugated with an EGFR antibody, were capable of recognizing EGFRvIII, which in turn was overexpressed in various human malignancies. EGFRvIII was reported to be overexpressed in the variability of human malignancies of epithelial origin, particularly in gliomas. As mentioned above, the reports indicated BNC’s potential as a means to achieve tumor targeting delivery [121].

The intravenous (i.v.) administration of T7 peptide modified core–shell NPs (T7-LPC/siRNA NPs) consisting of protamine/chondroitin sulfate/siRNA/cationic liposomes assembled layer by layer followed by modification using T7 peptide resulted in siRNA targeted delivery. T7-LPC/siRNA NPs, when compared with PEG-LPC/siRNA NPs, showed increased fluorescence intensity in microvascular endothelial cells of the brain (BMVECs) and U87 glioma cell lines. The NPs resulted in the downregulation of expression of EGFR protein in U87 glioma cells in vitro. The accumulation of NPs was more specific to the tumor tissues and penetrated the deep region ascertained by the co-culture model of BMVECs and U87 cells and in vivo imaging. The reports also confirmed that the NPs demonstrated the most prolonged survival period and highest down-regulated expression of EGFR, thereby showing the potential of siRNA delivery for the targeted therapy of GBM [122].

#### 7.1.2. Immunoliposomes (IL) and Solid Lipid Nanoparticles (SLNs)

The sustained and targeted drug release profiles of the immunoliposomes (ILs) and solid lipid nanoparticles (SLNs) nanosystems enabled the enhanced cancer cell inhibition and decreased the adverse effects throughout the tumor therapy [123]. Lipids (phospholipids) were utilized to manufacture NPs due to their safety and biocompatibility [124]. The dual targeting SLN loaded with etoposide (ETP) containing mAb for insulin receptors and anti-EGFR was used to treat GBM. The dual-functionalized SLNs crossed the BMVECs/HA (human astrocytes), which is an in vitro model for BBB, and showed enhanced cytotoxicity against U87MG cells [125], thus proving its potential against GBM. In another study, the Cetuximab (C225)-immunoliposomes (ILs) encapsulating boron anion were constructed by using novel maleimido–Polyethylene Glycol (PEG)–cholesterol for the targeted delivery of boron compounds to EGFR (+) glioma cells for boron neutron capture therapy (BNCT). It was concluded that the prepared ILs could serve as an efficient delivery vehicle for the BNCT of glioma [126].

Quantum dot immunoliposome (QD-IL), a hybrid nanoparticle, was targeted toward EGFR to treat GBM. QD-ILs were taken up efficiently by the malignant cells. In addition, QD-ILs served as imaging methods proven in both the in vitro and in vivo models. Furthermore, the NPs were also employed in ligand-directed delivery that allowed targeted drug delivery to the desired site to achieve efficient treatment for GBM [127]. Moreover, the anti-tumor effect of the combination of bevacizumab (Bev) and gemcitabine (GM) loaded IL (Bev-GM-IL) in a xenograft mice model (XMM) showed that the combinational therapy is better than monotherapy. This is due to the synergistic activity of two different drugs on GBM stem cells. Likewise, the combinational treatment extended the mean survival time of XMM. Altogether, the above results suggested that the combination of Bev-GM-IL offered promising outcomes in the treatment of GBM [128].

**Figure 5:**
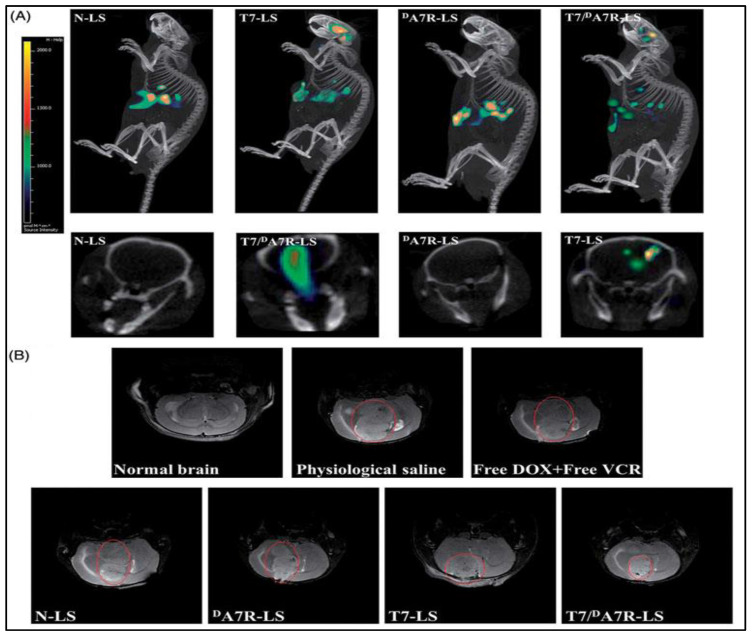
In vivo anti-glioma effect of doxorubicin (DOX) and vincristine (VCR)-loaded T7/^D^A7R-LS immunoliposomes. (**A**) Distribution of Cy5.5 in the mice brain bearing intracranial C6 glioma determined by a CLSM; (**B**) MRI of normal and pathological brains at 16 d after inoculation. Reprinted from [129], Taylor and Fransis Group, 2017.

In another study, doxorubicin (DOX) and vincristine (VCR) were loaded with T7 and ^D^A7R dual peptides-modified liposomes (T7/^D^A7R-Ls) to treat glioma. The in vivo (Figure 5) results of T7/^D^A7R-Ls showed improved glioma localization compared with mono ligand-modified liposomes or the free drug. In conclusion, the dual-targeting, co-delivery approach delivered a potential method for successful brain drug delivery in the glioma treatment [129].

#### 7.1.3. Polymeric Nanoparticles

The comparative evaluation of the other conventional nanocarriers, the polymeric NPs, exhibited promising benefits in the biomedical applications due to their improved solubility, biocompatibility, and biodegradability. The biodegradation through circulation in vivo can be eluded efficiently, and the elimination half-life of the drugs is also prolonged after polymeric NPs encapsulation [130]. Additionally, the polymeric NPs with an applicable particle size distribution could passively help accumulate medicines in the tumor region by improved permeability and retention time [131,132]. Moreover, the retention time of encapsulated drugs in the tumor region could also be precisely regulated by various strategies [133]. For all of these advantages, the surface of polymeric NPs could be altered by selecting specific ligands to achieve active targeting delivery [134]. A study demonstrated the delivery of curcumin using poly (D, L-lactic-co-glycolic acid) (PLGA) NPs tagged with an EGFRvIII, which was internalized by EGFRvIII overexpressed GBM cells leading to the enhanced photodynamic toxicity of curcumin [135].

Lei Wang et al. (2015) prepared the angiopep-2 (ANG)-modified PLGA/DOX/siRNA NPs, which inhibited the cells by inducing apoptosis and silenced the EGFR pathway in U87MG cells. The NPs were capable of penetrating the BBB, thus resulting in the enhanced accumulation of drugs in brain in vivo. Animal studies not only demonstrated the co-delivery of DOX and EGFR SiRNA but also prolonged the life span of GBM-bearing mice [136]. Chengkun et al. (2019) utilized the Golgi phosphoprotein 3 (GOLPH3) nanobody to construct an angiopep-2 (A2)-modified cationic lipid PLGA NPs (A2-N) targeting the Ge and GOLPH3 siRNA (siGOLPH3). The NPs not only penetrated the BBB but also silenced the expression of GOLPH3 mRNA and enhanced the expression of EGFR and pEGFR upon entering glioma cells. In addition, the above-mentioned NPs acted as a combinational anti-tumor therapy in vitro and in vivo [137]. In vivo imaging revealed that the T7-LPC/siRNA NPs penetrated the deeper regions of the tumor. Furthermore, the accumulation was more in the brain, which was an added advantage compared to PEG-LPC/siRNA NPs. The group also demonstrated the enhanced survival period by down-regulating the expression of EGFR in mice, and therefore, it can serve as a potential target for treating GBM [119]. In another study, C225 was conjugated to TMZ-loaded PLGA NPs (C225–TMZ–PLGA–NPs) by cross-linking chemistry to target the EGFR receptor. Furthermore, in vitro cellular uptake and the in vivo evaluation of PLGA–NPs, TMZ–PLGA–NPs, and C225–TMZ–PLGA–NPs were conducted. In addition, the results of cell cytotoxicity, apoptosis in U-87MG, SW480, and SK-Mel 28 cancer cell lines confirmed that the C225-PLGA-NPs can be utilized as a versatile nanocarrier for the management of EGFR overexpressing cancers [138].

#### 7.1.4. Dendrimers

The dendrimers are hyper-branched macromolecules that exhibit advantages over the conventional carriers (liposome, polymeric NPs etc.) such as enhanced stability in the blood circulation and ability to accommodate various ligands due to its chemosynthetic approach rather than self-assembly through non-covalent interaction [139,140]. In addition, the structure, size, and molecular weight of dendrimer have resemblance with bio-structures and proteins (insulin, hemoglobin and cytochrome), which makes them employable in various fields as gene delivery, immunodiagnosis, and encapsulation of drugs [141,142]. Dendrimer-based drug delivery employing polyamidoamine (PAMAM) was also explored for its application in GBM therapy [143,144].

An antisense oligonucleotide (ASODN) delivery of conjugates of folate–PAMAM (FA-PAMAM) inhibited the C6 cell growth in glioma. The coupling of folic acid to the surface amino groups of PAMAM dendrimers and ASODNs (ASODN: FA-PAMAM) corresponded to rat EGFR in the ratio of 16:1. The ASODN:FA-PAMAM combination suppressed EGFR and C6 cell growth expression, thus enhancing the survival time [145].

Cetuximab (C225) could be covalently linked to methotrexate (MTX) by the 5th generation (G5) of PAMAM dendrimers via its fragment crystallizable (Fc) region (C225–G5–MTX) to target EGFR and EGFR variant III (EGFRvIII). Competitive binding assay (CBA) demonstrated that C225–G5–MTX exhibited a higher affinity for the EGFR-expressing rat glioma cell line (F98_EGFR_) than the wild-type rat glioma cell line (F98_WT_). Subsequently, the improved distribution of ^125^I bio-conjugate of C225-G5-MTX noticed in F98_EGFR_ was six-fold greater than F98_WT_ cells, thereby contributing to specific molecular targeting the GBM treatment. The animal models that received C225-G5-MTX and C-225 or MTX exhibited 15- and 19.5-day survival rates, respectively. Correspondingly, the results were non-significant between the control and test animals [146].

PAMAM dendrimer and Tat peptide were fabricated to bacterial magnetic NPs (Tat–BMPs–PAMAM), which were then complexed with the siRNA expression plasmid of human EGFR (psiRNA–EGFR) through electrostatic interplay (Tat–BMPs–PAMAM/psiRNA-EGFR). The conjugate offered promising results in reducing tumor growth and suppressing the expression of oncoproteins. In addition, the conjugate could serve as a possible targeted gene delivery for GBM [147].

**Figure 6:**
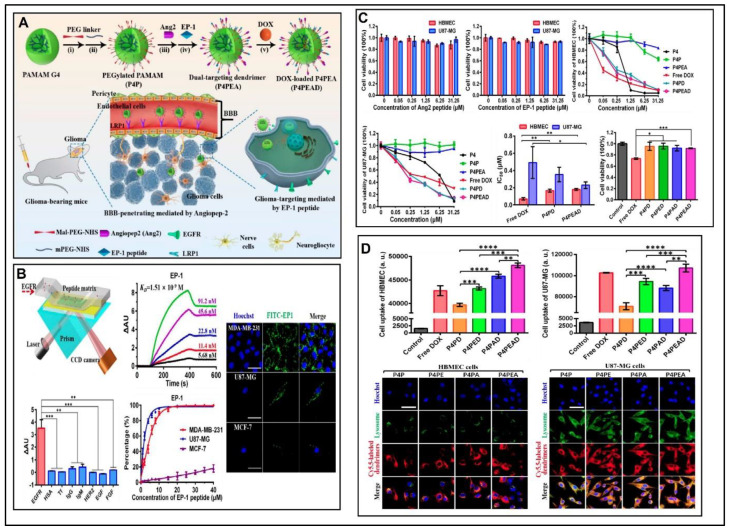
(**A**) Schematic representation of fabrication of the dual drug loaded polyamidoamine (PAMAM) dendrimers in the treatment of gliomas by improving BBB penetration; (**B**) Assessment of the affinity and specificity of peptide EP-1 toward EGFR; (**C**) In vitro evaluation of biocompatibility and anti-tumor efficacy of the dual drug-loaded PAMAM dendrimers; (**D**) Flow cytometry evaluation for intracellular uptake of different DOX-loaded dendrimers. Reprinted from [148] Ivyspring International Publisher, 2020. * *p* < 0.1, ** *p* < 0.01, *** *p* < 0.001, **** *p* < 0.0001 (Student’s *t*-test).

Recently, an angiopeptide-2 (Ang2) and low-density lipoprotein receptor-relative protein-1 (LRP1) was conjugated with PAMAM to improve BBB penetration glioma sites. Furthermore, PAMAM was concurrently functionalized with an EGFR-targeting peptide (EP-1) to achieve specificity and improved affinity to target EGFR. The above results showed the potential of the dual drug-loaded PAMAM in the treatment of gliomas by improving BBB penetration and specific EGFR targeting efficiency, both in vitro and in vivo (Figure 6) [148].

All the above-mentioned experiments concluded that the dendrimer-based NPs could be utilized as extensive drug delivery carriers to target and treat various CNS cancer cells by BBB penetration with the backing of targeting ligands.

### 7.2. Inorganic Nanoparticles (NPs)

#### 7.2.1. Silica NPs

The mesoporous silica nanoparticles (MS-NPs) are frequently employed as multifunctional nanocarriers to treat cancer cells owing to its mesoporous structure and enormous surface area. As a result, the active components can be dumped in the porous structure of NPs to obtain the maximum amount of drug-loading, and the surface modification of MS-NPs could also increase the intracellular uptake. Additionally, the alteration of carriers’ particle size, surface charge, and shape could increase the biocompatibility and minimize the cytotoxicity of MS-NPs [149]. In addition, MS-NPs could be encapsulated with contrast agents for MRI imaging. Hence, MS-NPs exhibit a chance to improve the solubility and stability of anti-EGFR drugs by being deposited in the porous structure.

**Figure 7:**
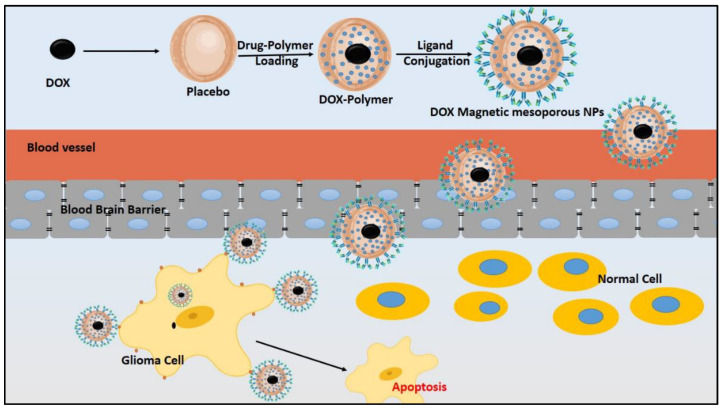
Synthesis of magnetic mesoporous nanoparticles using polymer and subsequently conjugated with ligands to achieve the sustained release of the drug at the specific targeted site.

Furthermore, prolonged drug release profiles resulted in decreased cytotoxicity in long-period cancer therapy [150,151]. The properties mentioned above were ensured by developing DOX magnetic (Fe_3_O_4_) NPs, encapsulated in polyethylene glycol (PEG), to functionalize the porous silica shell and treat cancer cells [152]. Likewise, multi-targeted oleic acid (OA)–MNPs were developed. Reports confirmed promising outcomes about the in vitro and in vivo efficacy in treating human cancer cells (HeLa). The study results stated that the MS-NPs formulations were more predominant than the placebo or free drugs and could overcome the drawbacks mentioned above of the conventional treatment approaches [153]. The synthesized and functionalized DOX magnetic MS-NPs were fabricated with PF-127 and then conjugated with transferrin (Tf) to enhance BBB penetration and achieve sustained release at the specific site. The Tf-loaded NPs resulted in improved BBB permeability (Figure 7). Thus, the prepared Tf nanocarriers could be considered as potential candidates in the treatment of brain tumors [154].

#### 7.2.2. Magnetic Nanoparticles (MNPs)

Magnetic nanoparticles (MNPs) specifically created an interest in the biomedical application and research due to various advantages: separation of molecules, gene/drug delivery, magnetic resonance imaging (MRI), and hyperthermic tumor treatment [155]. Among the several magnetic nanocarriers, super-paramagnetic iron oxide (SPIO) has been commonly utilized owing to its promising biocompatibility and magnetic properties. For example, an improved survival rate was noticed in animal models with C225-IONPs compared to pure C225 in the treatment of GBM [156]. In addition, SPIO and peptide nanoprobe were effectively combined and demonstrated for specific molecular MRI and sensitive optical imaging (SOI). Both in vitro and in vivo MRI and SOI showed that the nanoprobe was useful for targeting GBM with desirable biosafety [157]. In another study, MNPs were employed by convection-enhanced delivery (CED) in the brain to target the EGFRvIII xenografts GBM model. Then, MRI was conducted to evaluate brain targeting and the delivery of conjugated MNPs after CED. The accomplishment of a human clinical trial containing a direct injection of MNPs into recurrent GBM for thermotherapy proved the safety, efficacy, and feasibility in the patients [158]. In a recent report, pazopanib was loaded in MNPs, which stimulated the ultrasound’s drug release. The enhanced drug distribution in the non-small cell lung cancer (NSCLC) region resulted in improved therapeutic outcomes [159].

#### 7.2.3. Noble Metal Nanoparticles (NM-NPs)

Commonly, the Gold (Au) NPs are known as noble metal NPs, which were comprehensively studied for biomedical applications due to their lower toxicity, distinctive electronic, and optic properties that prompted cellular destruction with an application of radiation [160] or light [161]. In addition, AuNPs could be loaded with organic molecules (antibodies), which enhanced the accumulation of AuNPs within specific cancer tissues or lesions [162]. AuNPs were loaded with malondialdehyde-modified low-density lipoprotein (MDA-LDL) antibodies by distinct chemistries for drug recognition and capture from the biological system [163]. Furthermore, 40 nm AuNPs with mAb targeting the EGFR acted by random adsorption to treat oral squamous cancer. The antibody-loaded AuNPs accumulation into the tumor region enhanced cancer cell death by photothermal therapy [161]. In addition, the 5 nm AuNPs were surface modified with EGFR antibodies and functionalized using GM for targeting GBM cells [164]. In another study, Au nanocubes (AuNCs) with pH or temperature sensitivity were prepared for erlotinib or DOX encapsulation. Firstly, the drug-loaded AuNCs could accumulate more at the cancer tissue; the particular acidic microenvironment of cancer initiated erlotinib’s release, precisely. Finally, the controlled release of DOX by near-infrared (NIR) laser irradiation improved the therapeutic effect of AuNCs-based nano carrier in A431 cancer cell lines [165]. In addition, the in vivo activity of the Au–C225 conjugate resulted in a similar effect compared to free C225, concluding that the site-specific conjugation to the AuNP did not affect the biological action of the EGFR antibody, thereby signifying the value of the intended functionalization approach. The opportunity to yield accurate AuNP–Immunoglobuin G (IgG) conjugates creates novel paths to assay the Au–C225 conjugate for cancer therapy, either for sensitizing tumor cells to external radiation [166]. Based on the promising results of the developed NM-NPs provided a novel way for the delivery of chemotherapeutic and TKIs drugs.

## 8. Current Clinical Studies of Nanoformulations

After promising results of preclinical investigations, the organic and inorganic nanoformulations have entered the clinical trials to assess the tolerability, safety, pharmacokinetics, and efficacy for the treatment of GBM [167]. Table 3 summarizes the list of current clinical trials on EGFR loaded nanoformulation for the treatment of GBM. Firstly, the researchers have a newly developed EnGeneIC delivery vehicle (EDV). This inorganic nanocarrier exploits antibody-targeted, transporting active anti-cancer drugs into EGFR-expressing cancer cells (EnGeneIC, Lane Cove West, Australia). In a phase I/II study, the recurrent GBM adult patients were dosed of up to 5 × 10^9^ to determine the safety and possible dose of EGFR–EDV–DOX (NCT02766699) [168]. The effect of anti-EGFR targeted DOX loaded into C225-decorated Immunoliposomes (ILs) (C225–ILs–Dox) is being evaluated in a phase I clinical trial (NCT03603379) [169]. Analogously, DOX–trastuzumab consisting of PEGylated liposomes has completed a phase I/II clinical trial (NCT01386580). The phase I clinical study of cationic liposomes loaded with cancer suppressor gene p53 and TMZ (SGT–53–TMZ) was conducted to observe minimal side effects with a 6-month progression-free survival (PFS) and overall survival (OS) rate in patients with advanced solid tumors (NCT02340156) [170]. The phase I/II clinical trial was conducted to estimate side effects and a suitable dose of EGFR-bispecific antibody armed T cells (EGFR-Bi-T) in GBM patients’ treatment. In phase I trials, the patients received EGFR-Bi-T intrathecal (IT) injection twice per week for four weeks to determine the efficacy and toxicity profile. In phase II trials, the patients received EGFR-Bi-T IT twice weekly for four weeks and then i.v. over 15–30 min twice weekly for two weeks (NCT02521090).

## 9. Future Perspectives

The results of EGFR–TKIs drugs in clinical research showed that the molecularly targeted medicines combined chemotherapy could achieve the highest therapeutic outcome compared to free active components. However, the low specific inhibition and drug resistance during treatment were difficulties in developing targeted molecular active agents. Novel signaling transduction anti-cancer drugs based on modified therapy could minimize or overcome drug resistance. Remarkably, nanotechnology’s benefits for the currently available chemotherapeutic and TKI agents provided alternative strategies for improving therapeutic results: low solubility and BBB penetration, and prolonging the drug accumulation in the cancer region, decreasing the side effects triggered by non-specific distribution. The nanocarriers’ design has been moved forward via its technological upgradations, yet the nanoplatform fails to attain comprehensive clinical interpretation. Thus, the nanocarrier delivery approach is intended to assure better chances of success in a clinical trial [172].

Moreover, the clinical trial evidence (Table 3) was intended to determine the safety and efficacy of nanoformulations for the GBM treatment that have been happening since the beginning of the twenty-first century. Hence, the results of clinical studies have not yet been published, which contribute to paving the way for the clinical interpretation of nanotherapies for GBM. The researchers have recently considered utilizing natural compounds such as polyphenols and cannabinoids to target EGFR and its downstream pathway in various cancer cell lines [173,174,175,176,177,178]. The brain’s microenvironment, prominently advanced as per our understanding, targeting GBM cells with Janus kinases/signal transducer and activator of transcription proteins (JAK/STAT) inhibitors via combinational approaches could boost immunity with the reduced oncogenic effect of the GBM cancer cells [179,180].

## 10. Conclusions

The clinical trials of the nano-based formulations of chemotherapeutic and/or TKI drugs in combination are awaited. After an extensive literature search, it could be stated that novel approaches to treat GBM using mono or combinational therapy of polyphenols and anti-EGFR drugs to target multi signaling pathways (EGFR, JAK/STAT, and PI3K/AKT/mTOR) would help overcome the multiple failures of EGFR–TKI drug trials. Furthermore, the novel nano-based combinational therapy might positively reverse chemotherapeutic and TKI drug-induced resistance.

## Figures and Tables

**Table 1:** Approved small molecular tyrosine kinase inhibitors for cancer therapy [16,19].

Drugs	*IC*_50_ (nmol/L)	Targeting Receptor	Disease
Gefitinib (Iressa^®^)	14.6	EGFR	NSCLC, pancreatic cancer
Erlotinib (Tarceva^®^)	2		
Icotinib (Conmana^®^)	45		
Lapatinib (Tykerb^®^)	10.8, 9.2	EGFR, HER2	Breast cancer
Neratinib (Nerlynx^®^)	92, 59	EGFR, HER2	NSCLC, breast cancer
Afatinib (Gilotrif^®^)	0.5	EGFR, HER2 14	NSCLC, breast cancer, squamous cell carcinoma of the head and neck
Imatinib (Glivec^®^, Gleevec^®^)	600, 100	Abl, PDGFR, Kit SRC, PDGFR	CML, CMML, GIST
Dasatinib (Sprycel^®^)	<10		CML resistant to imatinib
Nilotinib (Tasigna^®^)	<30		CML resistant to imatinib
Sunitinib (Sutent^®^)	<100	VEGFR1-3, PDGFR, Kit FLT3, RET, CSF1R GIST, BRAF	Advanced RCC, CML resistant to imatinib, Hepatocellular carcinoma
Sorafenib (Nexavar^®^)	<100		
Pazopanib (Votrient^®^)	<150		

**Table 2:** Ongoing trials targeting the EGFR in glioblastoma (GBM).

Drug	GBM	Phase	Characteristics	NCT No.
Small-Molecule Kinase Inhibitors and/or Combination with Other Therapy				
GC1118	R	II	Focuses on overall response rate and exploration of predictive/prognostic biomarkers	NCT03618667
Osimertinib Fludeoxyglucose F-18 (FDG)	R	II	Studied the intra-patient variability of tumor FDG uptake, which was determined using double baseline FDG PET prior to osimertinib exposure	NCT03732352
EGFR BATs with SOC RT and TMZ	R	I	Immune measures in blood anti-GBM cytotoxicity of peripheral blood mononuclear cells directed at GBM cell lines	NCT03344250
Dacomitinib	C	II	Progression-free survival (PFS) at six months (PFS6m) and Safety and tolerability of oral administration of PF-00299804.	NCT01520870
Temozolomide, ABT-414, Radiation	A, NR	II III	Overall Survival (OS)	NCT02573324
EGFR(V)-EDV-Dox	R	I	Overall Survival (OS) and identification of recommended phase 2 dose of EGFR(V)-EDV-Dox in subjects with recurrent GBM	NCT02766699
C225-ILs-dox	R	I	Tumor response achieved in the treatment phase was assessed as per RANO criteria	NCT03603379
Protein expression analysis	C	-	Overall survival and the free survival was predicted based on the molecular characteristics	NCT00897663
EGFRvIII-CARs	R	I	Assessment of T cell trafficking within the brain tumor	NCT03283631
EGFRBi Armed Autologous T Cells	W	I II	Overall survival, change of cytokine profile, incident toxicity, and the overall survival was assessed as per the National Cancer Institute Common Terminology Criteria for Adverse Events Version 4.0	NCT02521090
Erlotinib hydrochloride	T	II	Disease response measured objectively by MRI of brain duration of progression-free survival (PFS)	NCT00387894
Radiation, temozolomide depatuxizumab mafodotin	A, NR	III	Cumulative dose of depatuxizumab mafodotin	NCT03419403
Gefitinib + Radiation therapy	C	I II	Overall survival by EGFR status	NCT00052208
Cetuximab, Mannitol	R	I II	Composite overall response rate was assessed through RANO	NCT02861898
AMG 596	R	I	Number of subject with treatment-emergent adverse events	NCT03296696
AMG 596	C	I	Overall survival and anti-AMG 595 antibody formation	NCT01475006
**PI3K/ART/mTOR**				
PX-866	C	II	Measurement of progression and response of brain tumor using MRI or CT scan	NCT01259869
INC280	T	I, II	Number of Patients Reporting Dose Limiting Toxicities (DLTs) in Phase 1 and Phase II Surgical Arm: Concentrations of INC280 and Buparlisib in Tumor	NCT01870726
XL765 (SAR 245409) + XL147 (SAR 245408)	C	I	To assess the biological effect and PI3K/mTOR modulations of XL 765 and XL 147 in GBM tissue	NCT01240460
BKM120 + Surgery	C	II	BKM120 brain plasma ratio at time of surgery	NCT01339052
MK-3475 + PI3K/AKT Inhibitors	#	I, II	Progression-free survival	NCT02430363
GDC-0084 + Radio Therapy	R	I	To estimate the maximum tolerated dose (MTD) or RP2D of GDC-0084 after radiation therapy (RT)	NCT03696355
AZD2014	A, NR	I	Recommended phase II dose (RP2D) of AZD2014	NCT02619864
GDC-0084	R	II	Dose-limiting toxicities (DLTs)	NCT03522298
AZD8055	C	I	Establishment of MTD of AZD8055 with recurrent gliomas	NCT01316809
GDC-0084 + Radiation Therapy	R	I	To estimate the MTD or RP2D of GDC-0084 after RT	NCT03696355
CC-115	A, NR	I	To determine the MTD, Non-tolerated dose and Dose-Limiting Toxicity	NCT01353625

R—Recruiting, C—Completed, A—Active, NR—Not Recruiting, T—Terminated, W—Withdrawn, and #—Study has passed its completion date and status has not been verified in more than two years.

**Table 3:** List of Current Clinical Studies of Nanoformulations.

Nano Carriers	Drug	Phase	Outcome Measures	NCT Number	Reference
EnGeneIC delivery vehicle (EDV)	EGFR-EDV-DOX	I	Determination of a possible phase II dose of drug for recurrent GBM.	NCT02766699	[168]
ILs	C225-IL-DOX	I	Determination of a suitable ratio of C225–IL–DOX concentration.	NCT03603379	[169]
PEGylated Lipososmes	DOX-Trastuzumab	I/II	To determine the safety and tolerability of i.v. administration of the PEGylated liposomes	NCT01386580	NA
Albumin NPs	Rapamycin + Avastin + Radiation	II	To determine progression-free survival (PFS) and overall survival (OS) rate according to response assessment in neuro-oncology (RANO) criteria	NCT03463265	[171]
Cationic Lipososmes	SGT-53 + TMZ	II	To determine six-month PFS and OS, anti-cancer activity, safety, and efficacy of NPs.	NCT02340156	[170]
enzyme-linked immune spots	EGFR-Bi-T	I/II	To determine the maximum tolerated dose (MTD) for eight intrathecal (IT) injections	NCT02521090	NA
